# Analysis of the factors affecting the evolution over time of subclinical hypothyroidism in children

**DOI:** 10.1186/s13052-016-0322-z

**Published:** 2017-01-03

**Authors:** Mariella Valenzise, Tommaso Aversa, Giuseppina Zirilli, Giuseppina Salzano, Domenico Corica, Simona Santucci, Filippo De Luca

**Affiliations:** Department of Human Pathology of Adulthood and Childhood, University of Messina, via Consolare Valeria, 98125 Messina, Italy

**Keywords:** Chromosomopathies, Down’s syndrome, Hashimoto’s thyroiditis, Thyroid function, Thyroid status, Turner syndrome

## Abstract

Aim of this commentary is to report the most recent views about natural history of subclinical hypothyroidism (SH) according to the different etiologies. In children with idiopathic SH the natural evolution is often favourable, with a high percentage of cases reverting to euthyroidism or remaining SH even after a prolonged follow-up. By contrast, the risk of a significant deterioration of thyroid status is distinctly higher in the SH children with Hashimoto’s thyroiditis (HT). This risk is even higher in the cases with both HT-related SH and chromosomal abnormalities, such as Turner or Down’s syndrome.

## Background

Subclinical hypothyroidism (SH) is a biochemical condition characterized by serum TSH concentrations above the upper limit of the reference range and serum FT4 levels within the reference range [1]. Although its prevalence is generally reported to be higher in the elderly population, this condition may be quite common even in children and adolescents and pediatric endocrinologists frequently face the decision of what to do regarding SH children [2].

Data concerning the natural course of this condition in pediatric patients are controversial, probably due to the fact that it may be significantly conditioned by the different etiologies. SH, in fact, may be possibly caused by the same thyroid diseases which result in overt thyroid function impairment and, particularly, Hashimoto’s thyroiditis (HT) [3, 4]. However, in most cases no definite etiology can be detected (idiopathic SH).

The aims of this commentary are to summarize the most recent views about natural evolution of SH in childhood and to analyze the most important predictive factors for its progression over time.

### Predictive factors for SH progression

The natural history of SH may be the reversion to euthyroidism or the persistence over time or the progression to frank hypothyroidism (with reduced FT4 levels).

In adults the risk of a progression toward overt hypothyroidism seems to be higher [5, 6] than that generally reported in children and adolescents [7–12]. In fact, in most of the available pediatric studies the rate of development of a frank hypothyroidism ranges between 0 and 28.8%, whereas the majority of initially SH patients revert to euthyroidism or remain SH [13]. The initial presence of goiter and elevated thyroglobulin autoantibodies, the coexistence of celiac disease and a progressive increase in the serum values of thyro-peroxidase autoantibodies and TSH may be predictive of evolution toward thyroid failure [12, 14].

However, from the analysis of pediatric literature, it emerges that baseline TSH levels are probably the most powerful predictors of SH evolution over time [10, 15]. In fact, children with high baseline TSH concentrations are likely to need supranormal amounts of TSH to adequately stimulate the thyroid and, therefore, the persistence over time of a SH status is not surprising in these children [10, 15].

Other factors which can play a significant role in conditioning the natural course of SH in children seem to be the etiology (either idiopathic or secondary to HT or obesity) and a possible association with either Turner syndrome (TS) or Down’s syndrome (DS), i.e., two chromosomopathies that are known to be linked with an increased risk of autoimmune thyroid diseases (AITDs) [16].

### Natural course of idiopathic SH

Pediatric studies on long-term evolution of SH in individuals with no apparent underlying thyroid disorders are few [10, 11, 17, 18].

In the retrospective and large study by Lazar et al. [10], TSH values during a 5-year follow-up tended to normalize over time, proportionally to the degree of TSH elevation at the onset of investigation. Predictive factors for a deterioration over time of thyroid function tests were an initial TSH value > 7.5 mIU/l and female gender, whilst age was not found to play a predictive role [10].

In the prospective multicenter study by Wasniewska et al. [11], based on a 2-year follow-up, 41.3% of the initially SH children normalized their TSH over time, whereas 58.7% remained SH and 12% increased TSH to >10 mIU/l. None of these children showed any symptoms of hypothyroidism during follow-up [11].

In the prospective study by Cerbone et al. [17], no alterations in growth, bone maturation, body mass index status and cognitive functions were observed, during a period of 2.0–9.3 years, in a series of children with idiopathic SH. These findings suggest that thyroid hormones involved in growth and neurocognitive development seem to be able to work properly, regardless of the persistently elevated TSH [2].

**Fig. 1:**
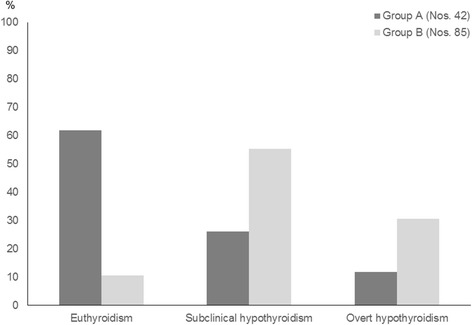
Prevalence (%) of the main biochemical pictures of thyroid function detected, at the end of a 5-year follow-up, in two groups of children who presented with either idiopathic subclinical hypothyroidism (SH) (Group A) or Hashimoto’s thyroiditis (HT)-related SH (Group B) (according to the results of Reference 18 study)

Finally, also the results of another five-year prospective study confirm that the natural history of idiopathic SH in pediatric age is characterized by a favourable long-term prognosis [18]. In fact, the majority of children with idiopathic SH (61.9%) spontaneously normalized over time their TSH values and only a minority (11.9%) became overtly hypothyroid at the end of follow-up (Fig. 1). By contrast, only a small minority (10.6%) of the patients who had presented with HT-related SH spontaneously normalized their TSH at the end of follow-up, whilst the majority remained SH or developed an overt hypothyroidism. On overall, these results confirm, on the basis of a prolonged prospective examination [18], the recent inference that underlying HT negatively affects the natural evolution of SH in children, irrespective of other concomitant risk factors [14].

As the majority of prospective pediatric studies demonstrate that idiopathic SH is often a remitting or self-limiting process, thyroid function monitoring in these children should not be performed too frequently: every 12 months, according to the recent guidelines of the European Thyroid Association [19].

A controversial issue in the management of children with idiopathic SH is whether these cases should be treated or not. The evidence for benefit of L-T4 supplementation is poor [15], although this does not necessarily imply lack of benefit [20]. If there is an underlying HT, treatment might be taken into consideration, since progression from SH to overt hypothyroidism is more likely in these cases [12, 14, 18].

### Natural course of HT- related SH

Thyroid function at HT presentation may significantly vary in the different pediatric cohorts [8, 9, 21–23]. In children euthyroidism is the most frequent presenting pattern, followed by SH and overt hypothyroidism [24]. Further and less frequent complaints of thyroid function which may be observed in children, at HT presentation, include either overt or subclinical hyperthyroidism [25, 26].

Thyroid function at HT diagnosis is mainly conditioned by patients’ age, with an increased risk of thyroid dysfunctions in the youngest children [4, 24].

In children and adolescents with HT, also the evolution over time of thyroid status may be quite variable [7, 9, 22, 27, 28] and is significantly affected by TSH serum levels at the time of HT diagnosis [7, 15].

According to the results of a recent report, a trend toward a progressive deterioration of thyroid status may be observed both in the initially euthyroid children and in those presenting with SH, although it has to be underlined that thyroid status prognosis in children with HT is not necessarily unfavourable [29]. In fact, at the end of a 5-year follow-up, 57.1% of the patients who had presented with euthyroidism remained euthyroid and 40.6% of those who were initially SH spontaneously normalized their thyroid function [29]. The results of that study, however, suggest that the patients presenting with SH may be more incline to develop over time a severe thyroid dysfunction picture, if compared with those presenting with euthyroidism [29].

In the light of the results of these studies on the natural course of thyroid status in children with HT- related SH, it might be proposed that thyroid function monitoring in these cases should be more strict than in children with idiopathic SH: every 6 months [30].

In the cases exhibiting a deterioration over time of thyroid status the hypothesis of a supplementation with L-T4 could not be preliminarily excluded, particularly considering the encouraging data reported by Svensson et al. [31]. These authors, in fact, in their retrospective study on 42 Swedish children with HT-related SH, found a significant reduction in median thyroid volume following a 2-year treatment with L-T4 [31].

### Natural course of HT-related SH in Turner syndrome (TS)

TS, that is one of the commonest chromosomal abnormalities, is known to be associated with an increased risk of developing AITDs and also celiac disease, type 1 diabetes, vitiligo and juvenile idiopathic arthritis [32–37].

In TS girls HT is by far the commonest autoimmune disease, with a relative prevalence which has been reported to be even more elevated than that generally reported in age-matched girls without TS [32, 34, 35]. Also the other one AITD, i.e., Graves’ disease (GD), has been reported to be distinctly more frequent in TS girls than in the pediatric general population [32, 38–40].

**Fig. 2:**
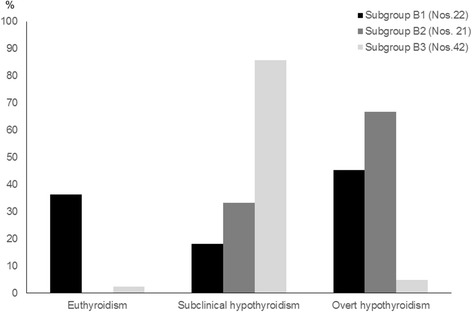
Prevalence (%) of the main biochemical pictures of thyroid function detected, at the end of a 5-year follow-up, in three subgroups of children with Hashimoto’s thyroiditis (HT)-related subclinical hypothyroidism without chromosomopathies (Subgroup B1) or with Turner syndrome (Subgroup B2) or Down’s syndrome (Subgroup B3) (according to the results of Reference 18 study)

The spontaneous evolution over time of thyroid function tests in TS girls with HT seems to be characterized by a significant worsening of thyroid status, both in the children presenting with euthyroidism and in those presenting with SH [41]. This spontaneous trend is especially evident in the TS girls with initial SH and is irrespective of both karyotype and other factors [42]. This inference was, just recently, supported by a 5-year prospective study [18], which confirmed that the association with TS is able to affect the course of HT, by increasing the risk of a thyroid function deterioration over time [18]. According to the results of that study [18], in fact, none of TS girls with HT-related SH reverted to euthyroidism, at the end of follow-up and the majority progressed to overt hypothyroidism (Fig. 2). Such an evolution pattern was distinctly more severe when compared with that recorded in the children with HT-related SH but without TS (Fig. 2).

To sum up, in the light of these peculiarities that characterize the natural course of TS-related HT, all individuals with TS should require continued monitoring of thyroid function throughout the life-span, as suggested by the TS Consensus Study Group [43].

### Natural course of HT-related SH in Down’s syndrome (DS)

DS is another relatively common chromosomopathy, which is known to be associated with an increased prevalence of AITDs [16]. In fact, both HT and GD may be encountered relatively often in the clinical history of DS children, probably due to a dysregulation of immune system, with secondary impairment of inhibitory activity [44]. This hypothesis could explain the common link with other extra-thyroidal autoimmune disorders [45, 46], which may be found in around 65% of DS children [47]. Among these diseases, the one that is most typically associated with DS is alopecia [48].

SH is common in DS children and should not be necessarily interpreted as consequence of HT. In fact, it might also result from a congenital alteration in the regulation of thyroid function [47], which has been reported to be peculiar of DS [49, 50].

Natural evolution of HT-related SH in DS children has been, just recently, investigated by means of a 5-year follow-up study [18]. In the light of the results of that prospective study, the association with DS might be able to condition a peculiar biochemical course of HT [18]. In fact, if the thyroid function biochemical patterns at the end of follow-up were compared with the ones found in the same DS children 5 years earlier, the almost totality of patients maintained a SH picture and only a minority of them reverted to euthyroidism or progressed to overt hypothyroidism [Fig. 2]. It is also noteworthy that, in 7.1% of DS children with HT-related SH, HT switched over time to GD [18]. This is not surprising, considering that DS children might be prone to manifest over time a phenotypic metamorphosis from HT to GD and to subsequently fluctuate from hypothyroidism to hyperthyroidism [51].

Finally, a further phenotypical peculiarity of DS patients is that clinical expression of autoimmunity in these individuals may be particularly severe [52].

### SH and obesity

Abnormalities of thyroid function are a frequent finding in obese children [53]. In particular, a SH may be encountered in 7.5% of these individuals [53]. The severity of SH in obese children is mainly conditioned by the level of overweight, whilst the distribution of patients with SH is not different between sexes and between prepubertal and pubertal individuals [53].

The pathophysiological mechanisms responsible for SH in obesity are not clear, although a role of leptin has been proposed [53]. In fact, serum leptin levels are increased in obese patients and some reports suggest that leptin can affect the hypothalamic regulation of TSH production [54]. However, since SH often reverts after weight loss, it seems to be a reversible complication of the overweight status and has not to be considered as a cause of obesity [53]. Therefore, any hormonal treatment should be avoided in obese children and also monitoring of thyroid function in these cases should not be too strict, since the risk of a progression toward overt hypothyroidism is not high. In fact, it has to be considered that FT3 serum levels are frequently increased in obese children, probably due to an adaptation process secondary to weight gain [53]. This should preserve obese children with SH from the risk of developing an overt hypothyroidism.

## Conclusions

Long-term prognosis of mild and idiopathic SH is frequently benign. The evidence for benefit of L-T4 supplementation is poor, although this does not necessarily imply lack of benefit. If there is an underlying HT, thyroid function monitoring has to be more strict, since progression from SH to overt hypothyroidism is more likely in these cases.

The association with either TS or DS furtherly impairs the outcome of HT-related SH.

## Abbreviations

AITDs: Autoimmune thyroid disorders
DS: Down’s syndrome
GD: Graves’ disease
HT: Hashimoto’s thyroiditis
SH: Subclinical hypothyroidism
TS: Turner syndrome
